# Pure androgen-secreting adrenal tumor (PASAT): A rare case report of bilateral PASATs and a systematic review

**DOI:** 10.3389/fendo.2023.1138114

**Published:** 2023-03-22

**Authors:** Zhangcheng Liao, Yuting Gao, Yang Zhao, Zhan Wang, Xu Wang, Jiaquan Zhou, Yushi Zhang

**Affiliations:** ^1^ Department of Urology, Peking Union Medical College Hospital, Chinese Academy of Medical Science and Peking Union Medical College, Beijing, China; ^2^ Department of Endocrinology, Peking Union Medical College Hospital, Chinese Academy of Medical Science and Peking Union Medical College, Beijing, China; ^3^ Department of Urology, Hainan General Hospital, Haikou, China

**Keywords:** pure androgen-secreting adrenal tumor (PASAT), clinical characteristics, diagnosis and treatment, adrenal venous sampling (AVS), ovarian venous sampling (OVS)

## Abstract

**Background:**

Adult pure androgen–secreting adrenal tumors (PASATs) are extremely rare, and their characteristics are largely unknown.

**Methods:**

A rare case of adult bilateral PASATs was reported, and a systematic literature review of adult PASATs was conducted to summarize the characteristics of PASATs.

**Results:**

In total, 48 studies, including 40 case reports and 8 articles, were identified in this review. Analysis based on data of 42 patients (including current case and 41 patients from 40 case reports) showed that average age was 40.48 ± 15.80 years (range of 18-76). The incidence of adult PASAT peaked at 21-30 years old, while that of malignant PASAT peaked at 41-50 years old. Most PASAT patients were female (40/42, 95.23%), and hirsutism was the most common symptom (37/39, 94.87%). Testosterone (T) was the most commonly elevated androgen (36/42, 85.71%), and 26 of 32 tested patients presented increased dehydroepiandrosterone sulfate (DS) levels. In malignancy cases, disease duration was significantly decreased (1.96 vs. 4.51 years, P=0.025), and tumor diameter was significantly increased (8.9 vs. 4.9 cm, p=0.011). Moreover, the androgen levels, namely, T/upper normal range limit (UNRL) (11.94 vs. 4.943, P=0.770) and DS/UNRL (16.5 vs. 5.28, P=0.625), were higher in patients with malignancy. In total, 5 out of 7 patients showed an increase in DS or T in the human chorionic gonadotropin (HCG) stimulation test. Overall, 41 out of 42 patients (including current case) underwent adrenal surgery, and recurrence, metastasis, or death was reported in 5 out of 11 malignant patients even with adjuvant or rescue mitotane chemotherapy.

**Conclusion:**

Adult PASAT, which is predominant in women, is characterized by virilism and menstrual dysfunction, especially hirsutism. Elevated T and DS may contribute to the diagnosis of adult PASAT, and HCG stimulation test might also be of help in diagnosis. Patients with malignant PASAT have a shorter disease duration, larger tumor sizes and relatively higher androgen levels. Surgery is recommended for all local PASATs, and Malignancy of PASAT should be fully considered due to the high risk of malignancy, poor prognosis and limited effective approaches.

## Introduction

The adrenal gland is a complex structure consisting of an inner adrenal medulla and an outer adrenal cortex. Adrenal steroid hormones are synthesized in the following three zones: aldosterone in the zona glomerulosa (ZG); glucocorticoids (particularly cortisol) in the zona fasciculata (ZF); and androgens and estrogens in the zona reticularis (ZR). Aldosterone production is regulated by the renin–angiotensin–aldosterone system, while the synthesis of cortisol and androgens is modulated by the hypothalamic−pituitary−adrenal (HPA) axis (1). The primary function of adrenocorticotropic hormone (ACTH) is to stimulate cortisol and adrenal androgen secretion by increasing their synthesis. The negative feedback from circulating cortisol suppresses the release of corticotropin-releasing hormone (CRH) and ACTH, but adrenal androgens do not have similar suppressive effects on the HPA axis. Compared to cortisol- and aldosterone-secreting tumors, androgen-secreting adrenocortical tumors are less common. Adrenocortical tumors, including androgen-secreting tumors, in children have been investigated in previous studies (2–4), but androgen-secreting adrenal tumors in adults, especially pure androgen–secreting adrenal tumors (PASATs), which are usually reported as case reports or case series, are rare. In the present study, we report a rare case of adult bilateral PASATs and conduct a systematic review of adult PASATs.

## Case presentation

A 28-year-old woman was admitted to our hospital in 2022 due to oligomenorrhea and then amenorrhea. The patient (menarche at 13 years) had regular menstruation with a menstrual cycle of 30-40 days and a menstrual period of 6-7 days. The patient’s menstrual cycle then shortened, and her menstrual bleeding gradually decreased from 2012. At that time, her testosterone (T) level was within the normal range according to her statement. Finally, amenorrhea occurred despite the administration of estrogen and progesterone drugs. In 2015, the T level increased to 0.91 ng/ml (normal: 0.10-0.75) despite dydrogesterone administration, and ultrasound indicated polycystic morphology in bilateral ovaries. Polycystic ovary syndrome (PCOS) was considered by physicians of other hospitals, and contraceptive drugs were prescribed. During 2019-2020, the patient presented with clitoromegaly, mild hirsutism (Ferriman–Gallwey score of 9) and amenorrhea. At the same time, bilateral adrenal tumors were reported incidentally by computed tomography (CT) in a health examination.

**Figure 1:**
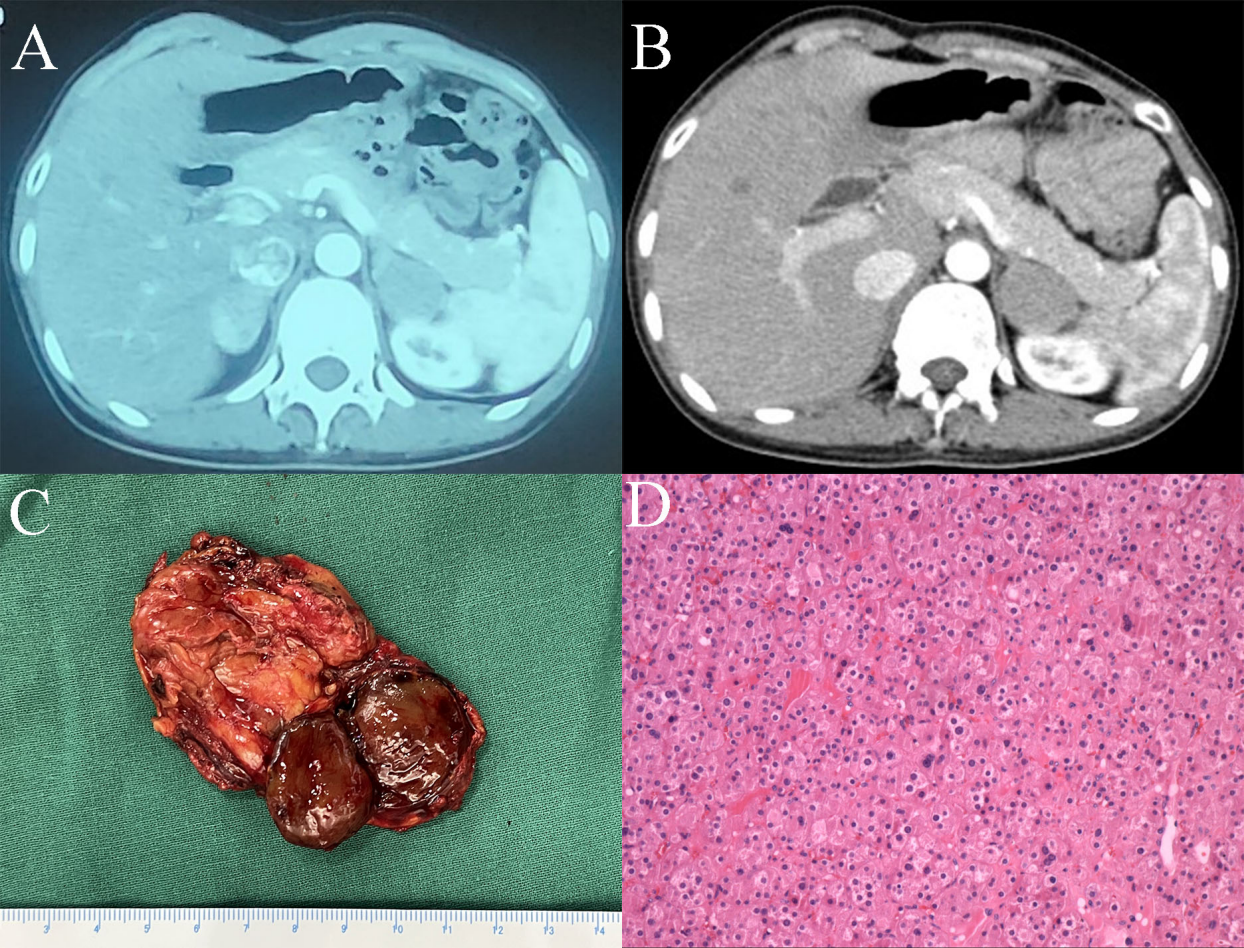
**(A)** CT image of bilateral adrenal tumors. **(B)** CT image of the left adrenal tumor after right adrenal tumorectomy. **(C)** Surgical specimen of the left adrenal tumor. **(D)** Haematoxylin and eosin staining of the left adrenal tumor (magnification×20).

An endocrine investigation in 2021 showed that the patient’s T levels continued to increase to 1.61 ng/ml (**Table 1**). In addition, androstenedione (AND), dehydroepiandrosterone sulfate (DS) and 17α-hydroxypregnenolone (17OHP) also increased to 25.67 ng/mL (normal: 0.30-2.00 ng/mL), 12,260 ng/mL (normal: 830-3,770 ng/mL) and 2.49 ng/ml (normal: 0.10-2.30 ng/ml), respectively, in the patient. The ACTH level (8 am) of this patient was 28.9 pg/ml (normal: 7.2-63.3). Both the cortisol level (10.0 µg/dl, normal: 4-22) and 24-h urinary free cortisol level (52.0 µg/24 hr, normal: 12.3-103) were normal. The cortisol level was suppressed (<1.8 µg/dl) by the 48-h low-dose dexamethasone-suppression test (0.5 mg orally every 6 hours for a total of eight doses). In addition, the levels of plasma aldosterone, catecholamine and sexual hormones, including follicle-stimulating hormone (FSH), luteinizing hormone (LH) and oestradiol, were normal. The patient underwent ACTH stimulation test, and the 17OHP level was 3.89 ng/ml at 60 minutes after administering cosyntropin (0.25 mg), which basically excluded classic and nonclassic congenital adrenal hyperplasia (CAH) according to the endocrine society clinical practice guidelines (17OHP <10 ng/ml) (5, 6). The patient’s bilateral adrenal tumors were gradually enlarged (**Figure 1A**) and were considered to be responsible for hyperandrogenism. The patient’s right adrenal tumor was removed by laparoscopic partial adrenalectomy at another hospital, and adrenocortical adenoma was identified according to pathological findings.

**Table 1:** Changes of androgens before and after two surgeries.

	T (ng/mL)	AND (ng/mL)	DHEA (ng/mL)	DS (ng/mL)
Before surgery	1.61	25.67	ND	12260
After right adrenal tumorectomy	1.01	6.44	23.8	11802
After left adrenalectomy	0.09	0.42	2.4	124
Reference range	0.08-0.60	0.30-2.00	<13	830-3770

T, testosterone; AND, androstenedione; DHEA, dehydroepiandrosterone; DS, dehydroepiandrosterone sulfate; ND, not detected.

**Figure 2:**
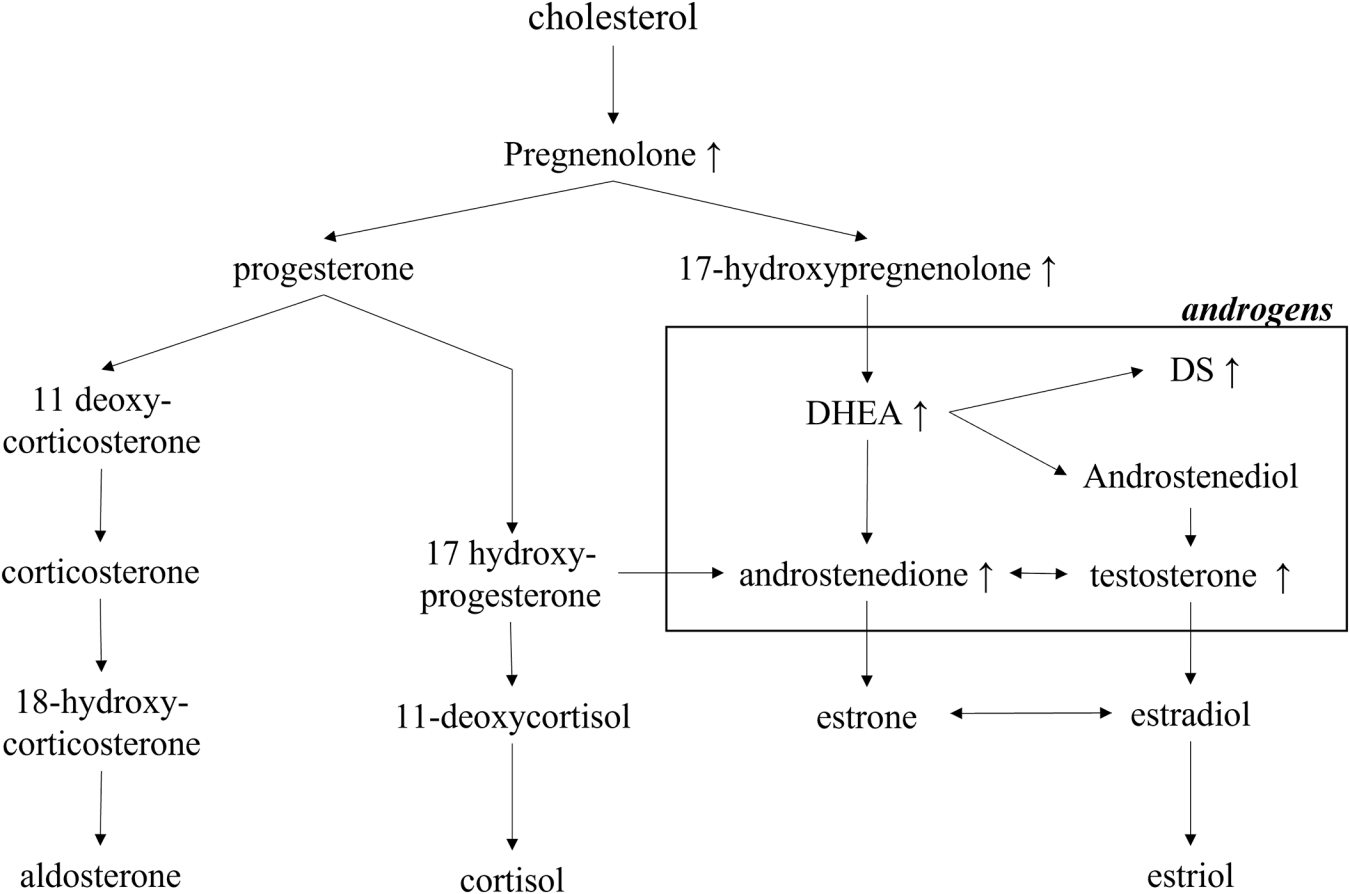
Exclusively increased steroid hormones in the androgen-producing pathway in the patient. DHEA, dehydroepiandrosterone; DS, dehydroepiandrosterone sulfate.

**Figure 3:**
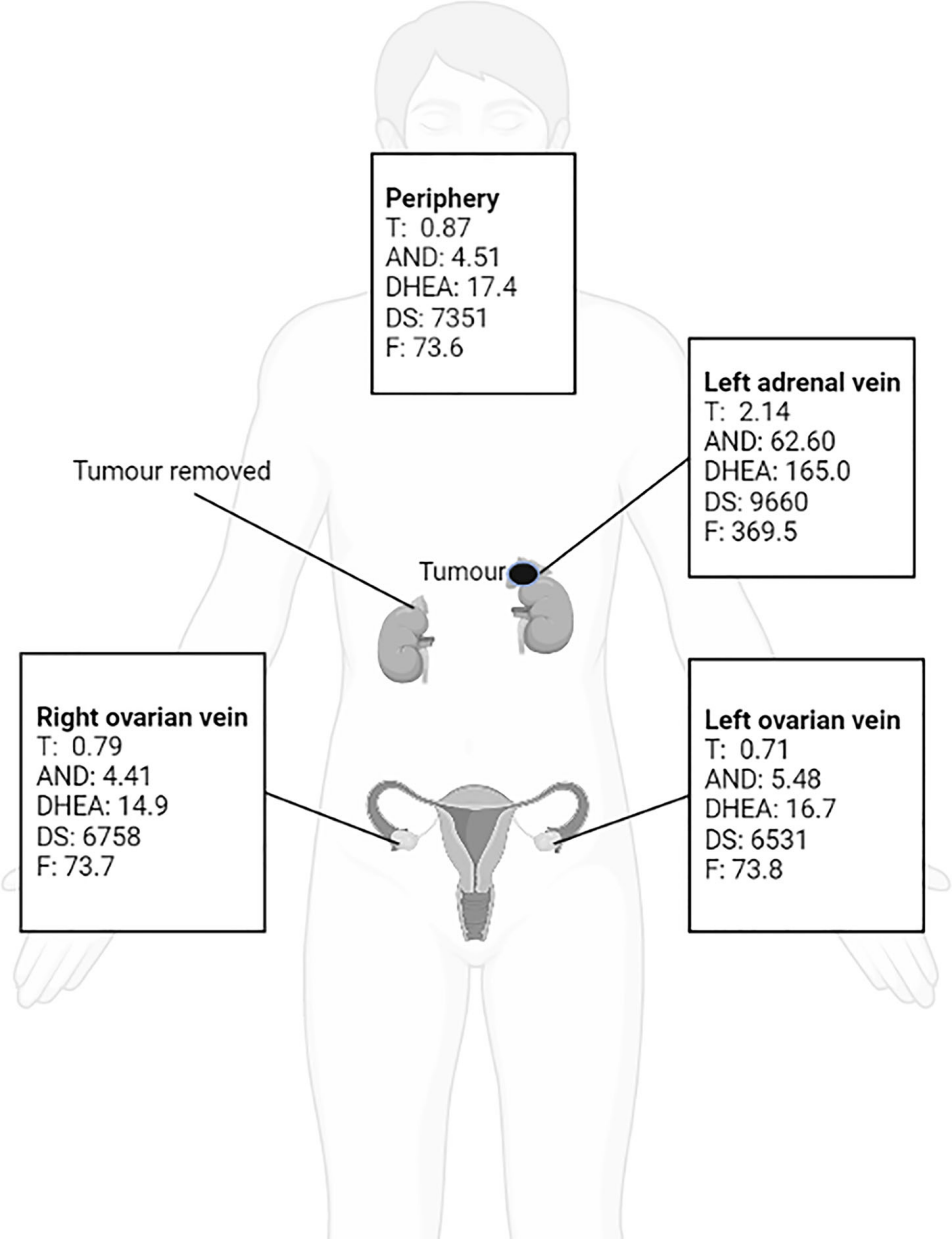
Bilateral ovarian venous sampling (OVS) and left adrenal venous sampling (AVS).

Because the patient’s amenorrhea was not relieved, she was admitted to our hospital and underwent further endocrine and imageological examination (**Figure 1B**). The patient’s testosterone (T), androstenedione (ADN) and dehydroepiandrosterone sulfate (DS) levels were partly decreased but still above the normal range (**Table 1**). Adrenal steroid hormone tests indicated that pregnenolone and dehydroepiandrosterone (DHEA) were also elevated in addition to T, DS, AND and 17OHP, while the levels of steroids located in the cortisol and aldosterone synthesis pathways were normal (**Figure 2**). Bilateral ovarian venous sampling (OVS) and left adrenal venous sampling (AVS) were conducted (**Figure 3**). The left selectivity index (SI, defined as the ratio of cortisol concentration for each adrenal vein and infra-adrenal inferior vena cava or a peripheral vein) was 5.02, which indicated successful catheterization (more than 2:1 without stimulation cosyntropin use) (7). The levels of androgens, including T, ADN, DS and DHEA, in the left adrenal vein were higher (**Figure 3**), which indicated that excessive androgens were produced by the left adrenal gland rather than the ovaries. The patient underwent left laparoscopic adrenalectomy in our hospital, and the surgical specimen was showed in **Figure 1C**. The histopathologic examination confirmed the diagnosis of adrenocortical adenoma (**Figure 1D**). Two weeks after surgery, the levels of T, ADN and DHEA decreased to within the normal range, and the DS level decreased to below the normal level (**Table 1**). Then DS level returned to the normal range within 3 months. The patient’s menstruation recovered to normal in 2 months after surgery, and no recurrence or metastasis was detected nearly one year after surgery.

## Methods

**Figure 4:**
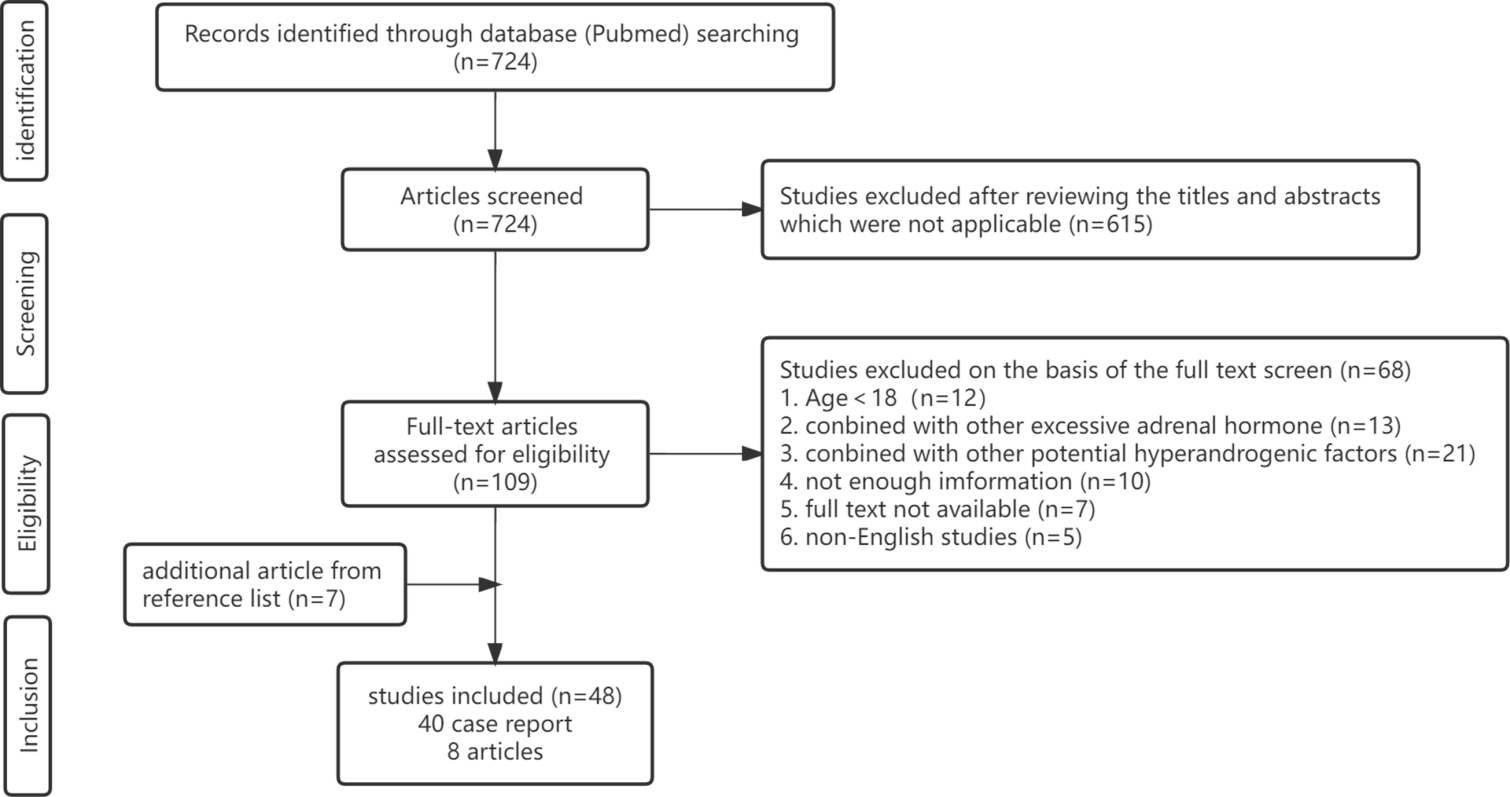
PRISMA flow diagram.

A systematic review of the literature was performed in accordance with the Preferred Reporting Items for Systematic Reviews and Meta-Analyses (PRISMA) guidelines (**Figure 4**). The following search strategy was used in the PubMed online database until December 2022: (“androgen”[Title/Abstract] OR “hyperandrogenism”[Title/Abstract] OR “virilism”[Title/Abstract]) AND (“adrenocortical”[Title/Abstract] OR “adrenal”[Title/Abstract]) AND (“tumor”[Title/Abstract] OR “carcinoma”[Title/Abstract] OR “adenoma”[Title/Abstract]) NOT (“children”[Title/Abstract] OR “child”[Title/Abstract] “childhood”[Title/Abstract] OR “paediatric”[Title/Abstract] OR “peripubertal”[Title/Abstract]). Only confirmed cases of adrenal tumors exclusively secreting androgens were included in this review. The exclusion criteria for studies were as follows: (1) age < 18 years; (2) excessive secretion of cortisol, aldosterone and catecholamine, especially cortisol, because cortisol and androgen are usually produced simultaneously in certain adrenal carcinomas or adenomas (only studies with evidence to show normal cortisol in the blood or urine were included); (3) hyperandrogenic status partly or completely caused by nonadrenal factors, including polycystic ovary syndrome (PCOS), idiopathic hyperandrogenism, hyperandrogenic insulin-resistant acanthosis nigricans syndrome (HAIRAN) and ovarian androgen-secreting neoplasms; (4) congenital adrenal hyperplasia (CAH); (5) basic science or animal experiments; (6) editorials, letters and consensus reports; (7) unavailable full text article; or (8) non-English studies.

The clinical data from the included studies were analyzed using GraphPad Prism (version 9; GraphPad Software, San Diego, California, USA).

Continuous data are presented as the mean ± standard deviation (SD), and Student’s t-test or the Mann−Whitney U test was utilized to compare the differences between the malignant and benign groups. A value of P<0.05 was considered statistically significant.

## Results

**Figure 5:**
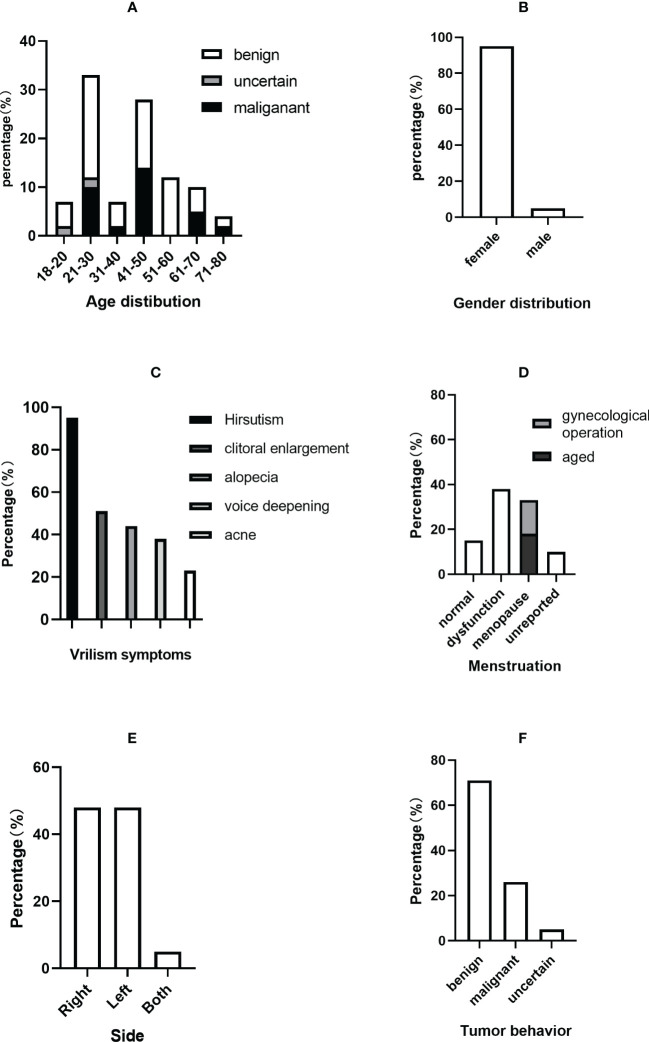
Clinical characteristics of PASAT cases. **(A)** Age distribution. **(B)** Sex distribution. **(C)** Virilism symptoms. **(D)** Menstruation change. **(E)** Tumor side. **(F)** Tumor behavior.

**Figure 6:**
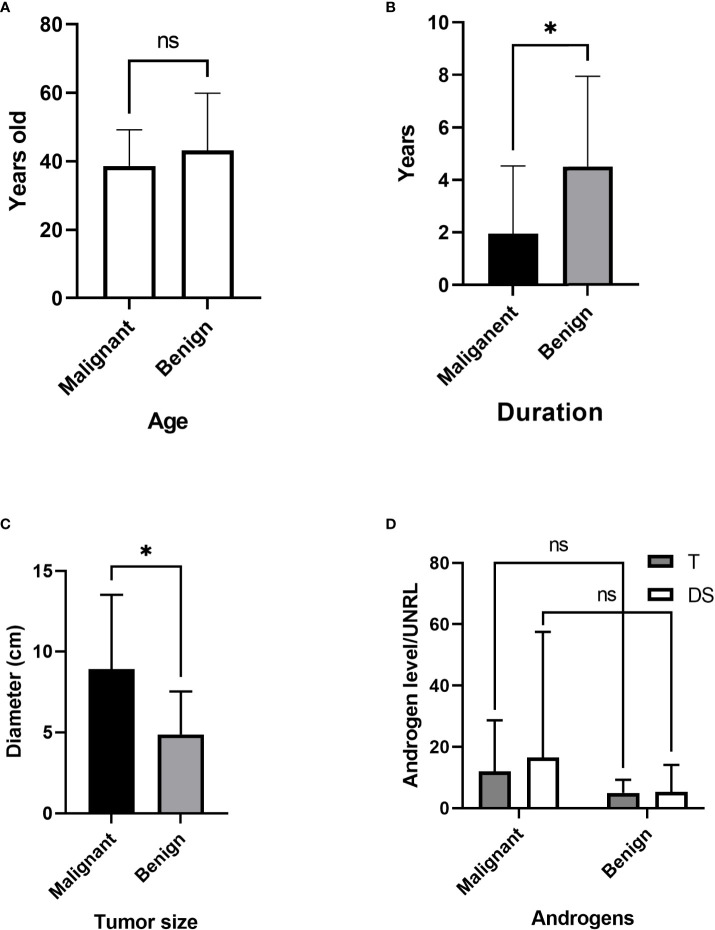
Comparison of malignant and benign tumors. **(A)** Age differences. **(B)** Duration before diagnosis. **(C)** Tumor size. **(D)** Androgen level. T, testosterone; DS, dehydroepiandrosterone sulfate; UNRL, upper normal range limit. ns, not significant; * P<0.05.

In total, 48 studies met the inclusion criteria, including 40 case reports (8–47) and 8 articles (48–55). Forty case reports including 41 adult PASAT cases are summarized in **Table 2**, and 8 articles containing adult PASAT patients are summarized in **Table 3**. Statistical analysis was conducted on the data of 42 PASAT adult patients (including the present case) (**Figures 5**, **6**).

**Table 2:** Characteristics of adult PASAT: summary of case reports (including current case).

case	year	1st author	Sex	Age(y)		Duration (y)	Hirsutism	Clitoromegaly	acne	voice deepening	alopecia	Menstruation	T/UNRL	DHEA/UNRL	DS/UNRL
1	1975	Blichert-Toft M	F	27		2	yes	–	yes	–	yes	normal	1.15	–	–
2	1976	Larson BA	F	76		3	yes	yes	–	–	yes	menopause (GS)	11.41	–	–
3	1977	Nogeire C	M	23		–	–	–	–	–	–	–	0.44	10.50	5.20
4	1978	Smith HC	F	50		5	yes	yes	yes	yes	yes	menopause (aged)	4.35	–	–
5	1980	Stephen W	F	62		3	yes	–	–	–	yes	menopause (aged)	5.49	–	–
6	1981	Trost BN	F	60		3	yes	–	yes	yes	–	menopause (aged)	11.54	–	–
7	1983	Vancouver	F	29		3	yes	yes	yes	yes	–	amenorrhea	2.47	–	1.95
8	1983	Fuller PJ	F	33		7	yes	yes	–	yes	–	normal	1.06	1.36	4.58
9	1983	Aguirre P	F	58		10	yes	yes	yes	yes	yes	menopause (aged)	3.35	–	–
10	1984	Faggiano M	F	20		–	yes	yes	yes	–	–	normal	1.39	–	2.54
11	1985	Vasiloff J	F	49		7	yes	–	–	yes	yes	menopause (GS)	14.08	0.15	–
12	1986	O'leary TJ	F	30		–	yes	yes	–	–	yes	normal	2.23	–	4.29
13	1986	Pollock WJ	F	60		4	yes	–	–	yes	–	menopause (GS)	8.13	0.21	–
14	1987	Guillausseau PJ	F	41		15	yes	–	–	–	–	amenorrhea	normal	increased	increased
15	1989	Clouston WM	F	45		7	yes	yes	–	–	yes	menopause (GS)	2.18	–	3.57
16	1991	Jeffrey A	F	64		8	yes	yes	–	–	yes	menopause (aged)	2.21	–	7.54
17-L	1992	Micić D	F	22		5	yes	–	–	–	–	amenorrhea	4.11	–	1.43
17-R															
18	1992	Ruutiainen K-patient 1	F	30		1	yes	yes	–	yes	–	normal	2.76	–	3.51
19	1992	Ruutiainen K-patient 2	F	41		1	yes	–	–	yes	yes	oligomenorrhea	2.28	–	0.49
20	1995	Coonrod DV	F	24		2	yes	yes	yes	–	–	normal	increased	–	normal
21	2000	Bozbora A	F	23		0.25	yes	–	–	–	–	amenorrhea	42.86	–	125.71
22	2007	Mavroudis K	F	45		2	yes	yes	–	yes	yes	–	1.78	–	0.66
23	2011	Amano T	F	31		0.16	virilism (no details provided)					amenorrhea	increased	–	–
24	2012	Surrey LF	F	55		–	yes	–	–	–	yes	–	14.11	–	1.58
25	2013	Rodríguez-Gutiérrez R	F	18		10	yes	yes	–	yes	–	amenorrhea	5.28	–	2.33
26	2013	Varma T	F	42		7	yes	yes	–	–	yes	amenorrhea	5.28	–	2.94
27	2013	Galketiya KP	F	47		–	yes	–	–	–	yes	irregular	3.40	–	1.90
28	2015	Tetsi Nomigni M	F	34		3	yes	–	–	–	–	irregular	2.70	–	0.17
29	2015	Uruc F	F	48		–	–	–	–	–	yes	–	1.19	–	5.30
30	2016	Ghorayeb NE	M	50		–	–	–	–	–	–	–	–	–	1.80
31	2016	Carré J	F	50		–	yes	yes	–	–	–	menopause (GS)	2.35	–	0.38
32	2018	LaVoie M	F	26		0.75	yes	–	yes	yes	–	amenorrhea	6.12	–	1.33
33	2019	Karimi F	F	26		1.5	yes	–	–	–	–	–	–	–	3.29
34	2019	Zhou WB	F	28		3	yes	yes	–	yes	–	amenorrhea	0.35	–	23.06
35	2019	Dotto RS	F	67		0.42	yes	yes	–	–	–	menopause (aged)	12.76	–	0.32
36	2020	Sailo SL	F	20		5	yes	yes	–	yes	–	amenorrhea	increased	–	–
37	2020	Pinge SR	F	57		–	–	–	–	–	yes	menopause (aged)	0.61	0.42	3.53
38	2021	Prachi	F	29		3	yes	–	–	–	–	irregular, amenorrhea	8.85	–	37.39
39	2021	Correia DN	F	41		0.83	yes	yes	–	yes	–	irregular	28.05	–	2.44
40	2022	Bao ZZ	F	23		2	yes	–	yes	–	–	irregular, oligomenorrhea	7.28	–	1.79
41	2022	Gopinath C	F	68		1.50	yes	–	–	–	yes	menopause (GS)	3.44	–	5.62
42-L	current case		F	28		7	yes	yes	–	–	–	amenorrhea	2.21	–	3.14
42-R	current case														

case	year	1st author	AND/ UNRL	location	Side	Tumor size(cm)	surgery	Pathology	biobehavioral	Outcome	Follow-up time
1	1975	Blichert-Toft M	–	US, angiography	R	8	tumor resection	adenoma	benign	well	3m
2	1976	Larson BA	normal	AVS, OVS	R	4	adrenalectomy	adenoma	benign	well	2m
3	1977	Nogeire C	–	abdominal exploration	R	–	tumor resection	carcinoma	malignant	recurrence, metastasis, dead	–
4	1978	Smith HC	–	–	R	3.5	adrenalectomy	adenoma	benign	–	–
5	1980	Stephen W	–	–	R	1	adrenalectomy	adenoma	benign	–	–
6	1981	Trost BN	–	AVS	R	3	adrenalectomy	adenoma	benign	well	2y
7	1983	Vancouver	0.88	CT	L	3	adrenalectomy	adenoma	benign	well	17m
8	1983	Fuller PJ	1.66	CT	R	5	tumor resection	adenoma	benign	–	–
9	1983	Aguirre P	–	abdominal exploration	R	3	adrenalectomy	ganglioneuroma	benign	–	–
10	1984	Faggiano M	0.86	X-ray, angiography	L	4	adrenalectomy	adenoma	benign	–	–
11	1985	Vasiloff J	0.46	CT	R	3	adrenalectomy	adenoma	benign	–	–
12	1986	O'leary TJ	–	CT	R	7.8	tumor resection	adenoma	benign	–	–
13	1986	Pollock WJ	–	CT	R	1.2	adrenalectomy	adenoma	benign	well	2y
14	1987	Guillausseau PJ	–	CT, AVS	L	10	adrenalectomy	adenoma	benign	well	6y
15	1989	Clouston WM	6.00	CT	L	6	tumor resection	adenoma	benign	well	3y
16	1991	Jeffrey A	–	CT	L	6	adrenalectomy	adenoma	benign	–	–
17-L	1992	Micić D	3.47	MRI, AVS, OVS	L	5.5	adrenalectomy	adenoma	benign	well	6m
17-R			–		R	5.4	adrenalectomy	adenoma			
18	1992	Ruutiainen	–	angiography	R	10.5	adrenalectomy	adenoma	benign	well	10m
19	1992	Ruutiainen	–	angiography	R	3.5	adrenalectomy	adenoma	benign	well	2.5y
20	1995	Coonrod DV	–	CT	R	6.4	adrenalectomy	carcinoma	malignant	well	8y
21	2000	Bozbora A	2.29	CT	R	9	adrenalectomy	carcinoma	malignant	metastasis, dead	8m
22	2007	Mavroudis K	1.21	MRI	L	2.5	tumor resection	adenoma	benign	–	–
23	2011	Amano T	–	CT, SPE-CT, PET-CT	L	5.8	adrenalectomy	carcinoma	malignant	well	1y
24	2012	Surrey LF	–	CT, MRI	L	7	tumor resection	oncocytoma, myelolipoma	benign	–	–
25	2013	Rodríguez-Gutiérrez R	3.70	CT	L	9.5	adrenalectomy	adenoma	benign	well	2m
26	2013	Varma T	–	CT	L	9.6	tumor resection	carcinoma	malignant	well	3m
27	2013	Galketiya KP	–	CT, PET-CT	R	10	adrenalectomy	carcinoma	malignant	well	1y
28	2015	Tetsi Nomigni M	3.47	CT, AVS, OVS	R	2.6	Laparoscopic adrenalectomy	oncocytoma	benign	well	3y
29	2015	Uruc F	–	US, MRI, PET-CT	L	1.7	tumor resection	carcinoma	malignant	mitotane ongoing.	–
30	2016	Ghorayeb NE	–	MRI	L	14.9	tumor resection	carcinoma	malignant	metastasis, dead	3y
31	2016	Carré J	5.84	CT	L	3.5	adrenalectomy	oncocytic carcinoma	malignant	well	4y
32	2018	LaVoie M	2.17	MRI	L	2.5	laparoscopic adrenalectomy	adenoma	benign	well	1y
33	2019	Karimi F	–	CT	L	15	non-resectable	carcinoma	malignant	metastasis, dead	–
34	2019	Zhou WB	–	CT	L	7.1	tumor resection	myelolipoma	benign	well	1.5m
35	2019	Dotto RS	0.73	ultrasound, CT, PET-CT	L	1.5	laparoscopic tumor resection	adenoma	benign	–	–
36	2020	Sailo SL	–	CT	R	7.1	adrenalectomy	adenoma	uncertain	well	2m
37	2020	Pinge SR	0.65	CT	R	3.8	adrenalectomy	adenoma	benign	well	11m
38	2021	Prachi	–	US, CT, PET-CT	R	8.1	laparoscopic adrenalectomy	oncocytoma	benign	–	–
39	2021	Correia DN	–	CT, PET-CT	L	13.2	extensive surgery	oncocytic carcinoma	malignant	metastasis, progressing	1m
40	2022	Bao ZZ	2.86	CT	L	10	adrenalectomy	oncocytic neoplasm	uncertain	well	4y
41	2022	Gopinath C	–	CT, PET-CT	L	7.3	laparoscopic adrenalectomy	adenoma	benign	well	7y
42-L	current case		7.78	CT, AVS, OVS	L	3.1	adrenalectomy	adenoma	benign	well	1y
42-R	current case				R	2.5	tumor resection	adenoma	benign		

T, testosterone; AND, androstenedione; DHEA, dehydroepiandrosterone; DS, dehydroepiandrosterone sulfate; UNRL: upper normal range limit; GS: gynecological surgery. US, ultrasound; AVS, adrenal venous sampling; OVS, ovarian venous sampling; CT, computed tomography; PET-CT, Positron emissiontomography–computed tomography. R, right; L, left.

**Table 3:** Summary of articles containing adult PASAT.

year	1st author	Time span(y)	Measured androgens	Adult PASAT (total subjects)	average age	Female (number)	average size	Bio behavior	Outcome
1988	Naganuma H	–	–	2 (19)	48.5	2	–	2 (B)	–
1993	Gaudio AD	1960-1990	T, DS, AND, 17-KS	8 (190)	41.6	–	–	6 (M)/2 (B)	4D, 2W (M)/ 2W (B)
1998	Filipponi S	1994-1997	–	1 (50)	–	–	–	1 (B)	–
2000	Wajchenberg BL	1982-1999	–	4 (47)	–	–	–	4 (M)	–
2003	Gordera F	1946-2002	T, DS, AND, 17-KS	7 (11)	24.6(M)/ 38.8(B)	7	10.7 (M)/ 4.2(B)	3 (M)/4 (B)	1D, 2W (M)/ 4 W (B)
2004	Moreno S	1970-2003	T, DHEA, DS, AND, 17-KS	Unknown*(21)	45(M)/ 34.5(B)	21	14 (M)/ 9 (B)	10 (M)/11 (B)	5D, 3R&MS, 2 W (M)/ 11W (B)
2011	Sarfati J	1995-2009	T, DS, AND	1 (22)	–	–	–	1 (M)	–
2017	Tong A	2002-2015	T, DS, AND	6 (9)	66(M)/ 32.2(B)	6	15 (M)/ 5.3 (B)	1 (M)/5 (B)	1 MS (M)/ 4W(B)

T, testosterone; AND, androstenedione; DHEA, dehydroepiandrosterone; DS, dehydroepiandrosterone sulfate; 17-KS; 17-ketosteroid; R, reoccurrence, M, malignant group; B benign group; MS, metastasis; W, well (no recurrence or metastasis observed in the following time or till death).

*the adult and preadult patients can’t be distinguished in this article and the youngest age of patient is 15 years old.

The average ages of all patients, the malignant group and the benign group were 40.48 ± 15.80, 38.64 ± 10.62 and 43.17 ± 16.73 years, respectively. The incidence of adult PASAT peaked at 21-30 years old, followed by 41-50 years old, while the incidence of malignant PASAT peaked at 41-50 years old (**Figure 5A**). In addition, PASATs were predominantly found in women (40/42, 95.23%; **Figure 5B**).

Hirsutism was the most common symptom, and almost all female PASAT patients (37/39, 94.87%) presented with hirsutism (one patient’s virilism was not described in detail), followed by clitoral enlargement, alopecia, voice deepening and acne (**Figure 5C**). Menstrual dysfunction, including oligomenorrhea, irregularity and even amenorrhea, was reported in 42.5% of female patients, while 15% of female patients showed normal menstruation (**Figure 5D**). There was no difference in tumor location (left or right side) (**Figure 5E**), but there were only two bilateral PASATs, including the present case. Tumors showing benign or malignant biological behavior accounted for 71.43% and 26.19%, respectively, with 2 uncertain tumors (**Figure 5F**).

The age differences between the malignant and benign groups were not significant (**Figure 6A**). The duration in the malignant group was significantly shorter (**Figure 6B**) than that in the benign group (1.96 vs. 4.51 years, P=0.025), while the tumor diameter in the malignant group was significantly increased (8.9 vs. 4.9 cm, p=0.011) (**Figure 6C**). For comparison, the androgen level was demonstrated as androgen concentration/upper normal range limit (UNRL). T was elevated in 36 adult PASAT patients (85.71%). The androgen levels, shown as T/UNRL (11.94 vs. 4.943, P=0.770) and DS/UNRL (16.5 vs. 5.28, P=0.625), in the malignant group were much higher than those in the benign group, but the differences were not significant (**Figure 6D**).

The responses of T and DS to the endocrine test are shown in **Table 4**. In the ACTH/synacthen test, the androgen levels increased in two patients but did not change in two patients. In another patient, the level of DS increased, but T was unresponsive. In the current patient, T decreased, but DS increased. The androgen response to the low-dose or high-dose dexamethasone test was not coordinated in different patients, and there were also paradoxical T and DS responses in the same patient. Regarding HCG (human chorionic gonadotropin) stimulation, most patients (5/7) showed an increase in DS or T. Furthermore, T and DS were slightly increased in the gonadotropin-releasing hormone (GnRH) test in two patients.

**Table 4:** Endocrine test summary of PASAT.

year	1st author	Age	Androgen(unit)	basal line	ACTH test	DXM test		HCG test	GnRH test
1975	Blichert-Toft M	27	T (ng/ml)	0.98	unresponsive	unresponsive (Low dose)	unresponsive (high dose)	Increased to 1.53 (5d, under DXM)	–
1978	Smith HC	54	T (ng/ml)	6.1-8	4.3→5.4(0.25mg over 8h, after DXM)	6.1 (2mgx7d)	4.4 (8mgx7d)	Increased to 10 (3000IUx4d)	–
1981	Trost BN	60	T (nmol/l)	22.2-27.7	–	29.8 (2mgx7d)	–	–	–
1983	Vancouver	29	T (ng/ml)	173	–	–	108 (8mgx3d)	–	–
			DS (ng/ml)	6600	–	–	6850 (8mgx3d)	–	–
1983	Fuller PJ	33	T (nmol/l)	–	–	unresponsive (dose unknown)	–	2.64→3.64 (5000IU over 16h)	4.4→5.6 (100mins)
1984	Faggiano M	20	T (ng/dl)	–	unresponsive (0.25mg)	decrease slightly (2mgx4d)	–	Increase (3000IUx3d, under DXM)	–
			DS (ng/ml)	–	unresponsive (0.25mg)	unresponsive (2mgx4d)	–	Increase (3000IUx3d, under DXM)	–
1986	O’leary TJ	30	T (nmol/l)	4.9	–	–	9.8 (8mgx2d)	unresponsive (5000IU)	–
			DS (nmol/l)	39.5	–	–	99.5 (8mgx2d)	unresponsive (5000IU)	–
1987	Guillausseau PJ	41	T (ng/ml)	0.56	1.98 (1mgx2d)	0.6 (2mgx2d, after HCG)	0.75 (8mgx2d, after low DXM)	0.9 (5000IUx3d, after ACTH)	–
			DS (ng/ml)	5100	13000	10000	8700	7400(5000IUx3d)	–
1989	Clouston WM	45	T (nmol/l)	6.6-8.7	–	9.4	14	–	–
			DS (μmol/l)	20-25	–	35	71	–	–
1992	Ruutiainen (case 1)	41	T (nmol/l)	–	–	–	unresponsive	–	–
			DS (μmol/l)	–	–	–	decrease slightly	–	–
1992	Ruutiainen (case 2)	30	T (nmol/l)	–	–	–	unresponsive (10mg injection)	–	–
			DS (μmol/l)	–	–	–	decrease slightly (10mg injection)	–	–
1992	Micić D	22	T (nmol/l)	–	–	20.2→15.2 (2mgx7d)	–	16.0→15.8 (3000IUx3d)	–
2007	Mavroudis K	45	T (pmol/l)	–	Unresponsive (0.25mg)	4.8→7 (0.5mgx7d)	–	–	–
			DS (μg/dl)	–	increased by 44%	2650→2200 (0.5mgx7d)	–	–	–
current case			T (nmol/l)	–	5.82→4.33 (0.25mg over 1h)	–	5.55→6.45 (2mgx2d)	–	6.66 (over 24h, after DXM)
			DS (μmol/l)	–	23.5→36 (0.25mg over 1h)	–	36.2→47.7 (2mgx2d)	–	66.4 (over 24h, after DXM)

ACTH, adrenocorticotropic hormone; DXM, dexamethasone; HCG, human chorionic gonadotropin; GnRH, gonadotropin-releasing hormone; T, testosterone; DHEAS, dehydroepiandrosterone sulfate.

The androgen level decreased in 36 patients in a short time after surgery, and virilism symptoms were also partly or completely relieved. However, details regarding androgens after surgery were not reported in six patients. All ten patient with malignancy underwent surgery, except for one patient with nonresectable disease with multiple metastases at diagnosis (**Table 2**). Mitotane was applied to seven malignant patients (5 post-surgically, 1 with nonresectable disease and 1 after recurrence). Five patients with malignancy (including the one with nonresectable disease) were reported to have experienced recurrence, metastasis, or death.

The articles containing adult PASAT were summarized **Table 3**. T and DS were the most frequent androgens used for hormone evaluation, and urinary 17-ketosteroid was a significant indicator of hyperandrogenism in the earlier studies. The age distribution of the patients with malignant or benign tumors varied among the different studies. The age of the malignant group was younger than that of the benign group in the study by Gordera F but older in the study by Moreno S. The average size of the malignant tumors was larger than that of the benign tumors. Most malignancy patients (14/20) had a negative outcome (recurrence, metastasis or death), while no patient with benign conditions were reported to have a negative outcome during the follow-up period.

## Discussion

Androgen-secreting adrenal tumors are rare. A recent population-based study of 1287 patients diagnosed with adrenal tumors reported only one androgen-excess adrenal tumor (0.1%) (56). In another study of 1205 patients with androgen excess, 20 (1.7%) individuals were identified as having androgen-secreting adrenal tumors (57). Androgen-secreting adrenocortical tumors, especially adult PASATs, remain a challenge in clinical diagnosis and treatment due to their rarity. Here, we report a rare case of adult bilateral PASATs, and to our knowledge, only one other case of bilateral PASATs has been reported since 1970. Furthermore, we conducted a systematic review of adult PASATs to increase the understanding of adult PASATs and provide guidelines for the diagnosis and treatment of adult PASATs.

The incidence peak for adult PASAT occurred at 21-30 years of age, while the incidence peak for malignant PASAT occurred at 41-50 years of age. Hirsutism, as one of the initial complaints, was the most common symptom found in the present review and other PASAT studies (53, 55, 58). Young females might focus more on their appearance, and they may notice hirsutism once it occurs, prompting them to visit doctors and undergo medical screening for virilism-causing adrenal tumors. This may contribute to the peak incidence of adult PASAT at 21-30 years of age. The incidence of malignant PASAT peaked at 41-50 years of age, which is close to the age of incidence peak of adult adrenocortical carcinoma in a previous study (51). Several factors may be responsible for this. Above all, the incidence peak of malignant PASAT may be associated with age because the risk of malignant tumors is higher in older people (59). It may also be associated with menstrual dysfunction. It was indicated that the occurrence of menstrual dysfunction is associated with higher androgen levels in women with hirsutism (60), and our data analysis demonstrated that patients with malignant PASAT presented with higher androgen levels, which indicates that malignant PASAT might lead to more menstrual disorders. It is well known that females aged 41-50 years usually begin to suffer menstrual problems due to perimenopause, prompting them to seek medical advice and hormone evaluation. Therefore, androgen abnormalities that are related to potential virilism-causing malignant adrenal tumors are more likely to be diagnosed in patients aged 41-50 years. However, the potential factors responsible for the age distribution of PASAT need to be identified in further research.

In the present review, PASAT was predominant (40/42) in women. The low incidence of PASAT in men may be associated with clinical ignorance. It is difficult to ascertain the onset of androgen-excess symptoms in males, which are usually clinically ignored until the presence of a consistent peripheral androgen-to-estrogen conversion is determined (61). In addition to hirsutism and menstrual dysfunction, other virilism manifestations, such as clitoral enlargement, alopecia, voice deepening and acne, were found to be important signs for screening and diagnosis.

Malignant adrenal neoplasms were identified in 26% of adult PASAT patients, which was significantly higher than the incidence (8.6%) of malignant adrenal tumors in an adrenal tumor population (56). The average duration before diagnosis in the malignant group was significantly shorter than that in the benign group, which may be related to the rapid progression of the malignant adrenocortical tumor. The malignant adrenal tumor size was significantly larger than that of benign tumors, consistent with various studies on adrenal tumors. Testosterone is the most sensitive marker in the hormone evaluation of PASAT, as it is elevated in almost all PASAT patients and is the only increased androgen in certain patients (41, 62, 63). Some studies (64, 65) have demonstrated that increased DS (produced uniquely by the adrenal zona reticularis) and DHEA (produced mainly by the adrenal zona reticularis) are indicators of adrenal etiology, especially DS. In the current review, however, the DS level was normal in 6/32 patients, and the DHEA levels was normal in 3/6 patients. The levels of T and DS, which are presented as androgen levels/upper normal range limit in **Table 2**, were much higher in malignant cases, but these differences were not significant and the potential differences in androgens between malignant and benign PASAT need to be identified in future studies due to the limited number of cases in the present review.

The contribution of the ACTH/synacthen test and dexamethasone test (low dose or high dose) to the diagnosis of PASAT may have been limited because the T and DS responses varied among different patients. However, most female patients showed an increased T and DS level in the HCG test, which may potentially contribute to the diagnosis of PASAT. Leydig cells or features related to Leydig cells, which could increase testosterone secretion under the stimulation of HCG, have been reported in the pathological findings of PASAT neoplasm (11, 13, 16, 18), and this may be the possible reason why PASAT responds actively respond to HCG.

A previous study indicated that as many as 45% of female patients with androgen-producing adrenal neoplasms underwent needless ovarian surgical treatment before obtaining the correct diagnosis (66). Polycystic ovarian morphology, a potential factor in hyperandrogenemia, has been reported in several PASAT patients (11, 24, 28, 38, 39) and was present in the current case, but it was identified as an irrelevant factor. Unfortunately, two PASAT patients included in the current review also underwent an unnecessary oophorectomy (11, 28). It is unclear whether polycystic ovarian morphology is caused by hyperandrogenemia itself, but it does lead to difficulties in identifying the source of hyperandrogenism in terms of location. Notably, adrenal venous sampling (AVS) or ovarian venous sampling (OVS) played a significant role in accurately identifying the aetiological location in some PASAT patients (9, 13, 21, 24, 34) and in the current case. Thus, AVS and OVS are recommended for the identification of the location of hyperandrogenemia aetiology, especially when clinical findings are inconclusive.

## Conclusion

Adult PASATs are extremely rare, and their characteristics are largely unknown. The diagnosis and treatment of adult PASATs remain a clinical challenge. Adult PASATs are predominant in women and most frequently characterized by hirsutism symptoms and other virilism signs. Serum testosterone is the most sensitive indicator, and elevated DS could contribute to the diagnosis of PASAT specifically. HCG stimulation test might also be of help in diagnosis. Individuals with malignant PASATs have a shorter disease duration before diagnosis, larger tumor sizes and relatively higher androgen levels. AVS and OVS may be important approaches for the location diagnosis. Surgery is recommended for all patients with local PASATs, whether benign or malignant. Tumor malignancy should be fully considered in patients PASATs due to the high risk of malignancy, poor prognosis and limited effective treatment approaches.

## Data availability statement

The raw data supporting the conclusions of this article will be made available by the authors, without undue reservation.

## Author contributions

Conceptualization, ZL, YG, and YSZ. Methodology, ZL and YZ. Validation, ZW and XW. Data analysis, ZL, YG, and JZ. Writing—original draft preparation, ZL. Writing—review and editing, YSZ. All authors contributed to the article and approved the submitted version.
